# Targeting SUMOylation in *Plasmodium* as a Potential Target for Malaria Therapy

**DOI:** 10.3389/fcimb.2021.685866

**Published:** 2021-06-10

**Authors:** Daffiny Sumam de Oliveira, Thales Kronenberger, Giuseppe Palmisano, Carsten Wrenger, Edmarcia Elisa de Souza

**Affiliations:** ^1^Department of Parasitology, Institute of Biomedical Sciences at the University of São Paulo, São Paulo, Brazil; ^2^Department of Internal Medicine VIII, University Hospital Tübingen, Tübingen, Germany

**Keywords:** malaria, *Plasmodium falciparum*, SUMO, SUMOylation, drug target

## Abstract

Malaria is a parasitic disease that represents a public health problem worldwide. Protozoans of the *Plasmodium* genus are responsible for causing malaria in humans. *Plasmodium* species have a complex life cycle that requires post-translational modifications (PTMs) to control cellular activities temporally and spatially and regulate the levels of critical proteins and cellular mechanisms for maintaining an efficient infection and immune evasion. SUMOylation is a PTM formed by the covalent linkage of a small ubiquitin-like modifier protein to the lysine residues on the protein substrate. This PTM is reversible and is triggered by the sequential action of three enzymes: E1-activating, E2-conjugating, and E3 ligase. On the other end, ubiquitin-like-protein-specific proteases in yeast and sentrin-specific proteases in mammals are responsible for processing SUMO peptides and for deconjugating SUMOylated moieties. Further studies are necessary to comprehend the molecular mechanisms and cellular functions of SUMO in *Plasmodium*. The emergence of drug-resistant malaria parasites prompts the discovery of new targets and antimalarial drugs with novel mechanisms of action. In this scenario, the conserved biological processes regulated by SUMOylation in the malaria parasites such as gene expression regulation, oxidative stress response, ubiquitylation, and proteasome pathways, suggest *Pf*SUMO as a new potential drug target. This mini-review focuses on the current understanding of the mechanism of action of the *Pf*SUMO during the coordinated multi-step life cycle of *Plasmodium* and discusses them as attractive new target proteins for the development of parasite-specific inhibitors and therapeutic intervention toward malaria disease.

## Introduction

The parasitic disease malaria represents one of the most serious threats to human health with an enormous impact on mortality and morbidity. According to the World Health Organization (WHO) reports that in 2019, there were 229 million cases and an estimated number of malaria deaths at 409,000, worldwide, of the estimated victims, the most vulnerable group affected by the disease are children below five years old. It must be emphasized that Africa holds 94% of these cases and deaths. Malaria in humans is caused by apicomplexan parasites of the genus *Plasmodium*, being the majority of recurrence due to five species: *Plasmodium falciparum, P. vivax*, *P. malariae, P. ovale*, and *P. knowlesi* (Sato, 2021). It is noteworthy, infection by *P. falciparum* (*Pf*), causes the most virulent form of human malaria (Cowman et al., 2017).

*Plasmodium* has a multistage life cycle that comprises specialized asexual form, it establishes infection and proliferation within the vertebrate host and sexual form, that occurs in the mosquito vector (de Jong et al., 2020). The distinct cellular environments during the stages of the *Plasmodium* life cycle require elaborated adaptation such as the use of post-translational modification (PTM), which provides a way for spatiotemporal control of cellular activity. Several PTMs are described in *Plasmodium* ssp. such as SUMOylation (also termed ‘SUMO conjugation’), ubiquitination, phosphorylation, acetylation, nitrosylation, lipidation, and methylation (Artavanis-Tsakonas et al., 2006; Issar et al., 2008; Chung et al., 2009; Ponts et al., 2011; Doerig et al., 2015; Li et al., 2021). SUMOylation emerges as an important PTM in the *Plasmodium* life cycle.

SUMOylation is an evolutionarily conserved PTM in which a small ubiquitin-like modifier (SUMO) modulates a wide variety of biological and molecular processes such as protein-protein interactions (Bayer et al., 1998) across several organisms such as parasitic protozoa, fungi, plants, humans, and others (Flotho and Melchior, 2013; Gupta et al., 2020). SUMO is a 12 kDa polypeptide found ubiquitously in the eukaryotic kingdom and holds structural and evolutionarily similarities to ubiquitin (UB) (Bayer et al., 1998). Classically, SUMO conjugation consists of the covalent linkage of SUMO protein on lysine residues of a protein substrate, which is triggered by the sequential action of three enzymes: heterodimeric E1-activating enzyme (Aos1/Uba2), E2-conjugating enzyme (Ubc9), and SUMO-E3 ligase, in an ATP-dependent manner (Streich and Lima, 2014; Pichler et al., 2017; Varejão et al., 2020). SUMO-conjugated maintains the fine-tuning of cellular functions by altering intracellular compartmentalization or modulating the protein stability, enzymatic activity, and interactions affinity (Moreno-Oñate et al., 2020; Sohn et al., 2021).

Novel treatments and, consequently, new therapeutic targets must be ready to overcome divergent biology associated with the complex life cycle stages of *Plasmodium* ssp. Further studies are necessary to precisely understand the molecular and cellular functions of *Pf*SUMO. In this minireview, we consider the potential of the SUMOylation pathway as a target for malaria disease intervention and highlight the current knowledge about the SUMO mechanism during *Plasmodium*’s life cycle.

## The SUMOylation Machinery in *Plasmodium*

Whereas the human genome carries five SUMO isoforms (Liang et al., 2016), comparatively *Plasmodium* encodes a single SUMO paralogue so far revealed in *P. falciparum*, namely *Pf*SUMO (Issar et al., 2008; Reiter et al., 2016). Bioinformatic approaches and *in vitro* biochemistry analysis of *Pf*SUMO conjugation indicated that the heterodimeric E1-activating enzyme (composed by Aos1/Uba2, herein referred to as *Pf*SUMO-E1) and the E2-conjugating enzyme (Ubc9 or *Pf*SUMO-E2) are functionally active in *P. falciparum* (Issar et al., 2008; Ponder et al., 2011; Reiter et al., 2013; Reiter et al., 2016). In this respect, *Pf*SUMO-E1 and *Pf*SUMO-E2 could interact and mediate SUMOylation of mammalian RanGAP1, a SUMO-substrate model (Reiter et al., 2013). Besides, *Pf*SUMO-E1 was able to activate both the *Pf*SUMO and the human SUMO-2 (Reiter et al., 2013; Reiter et al., 2016).

**Figure 1:**
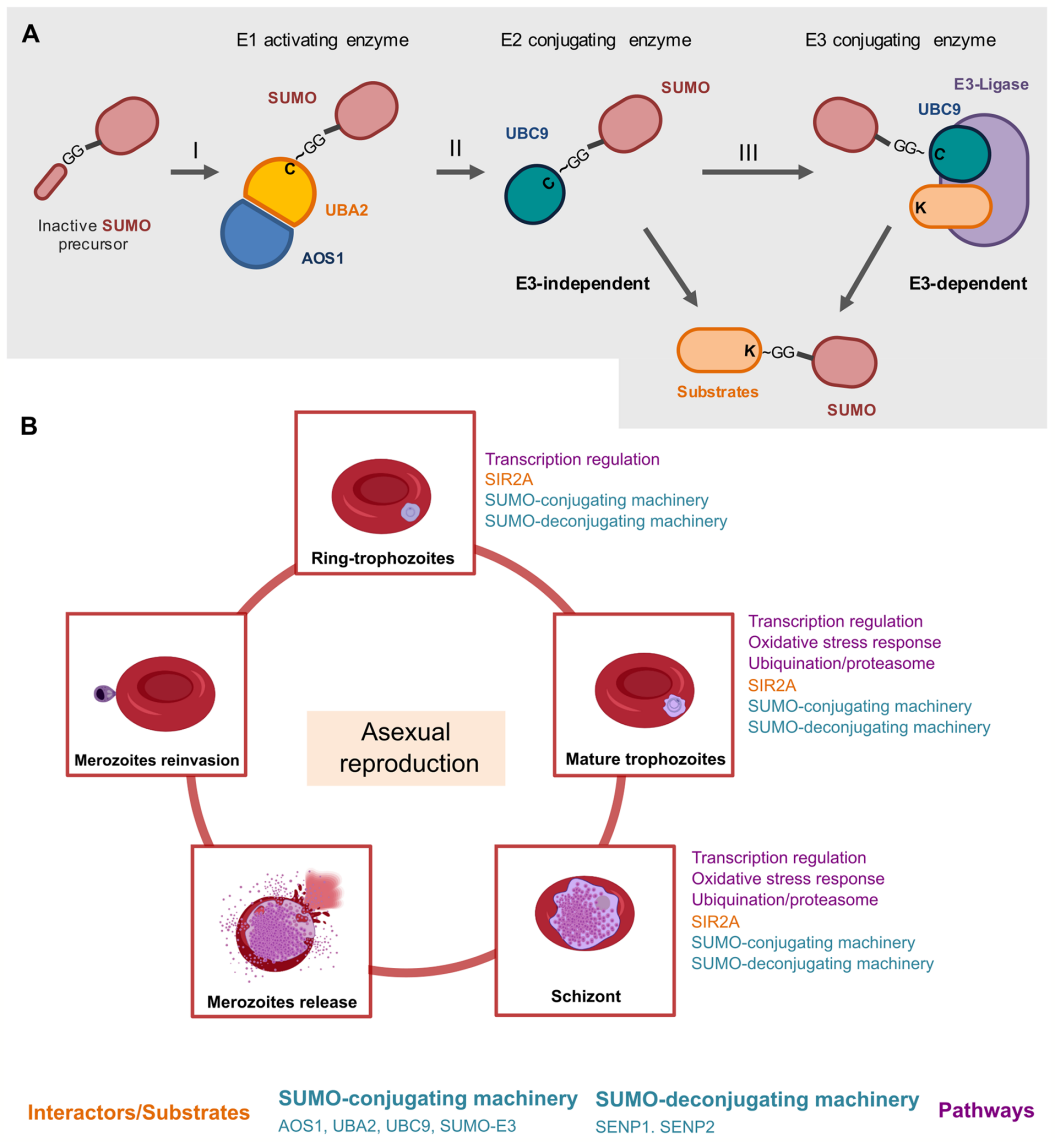
A schematic overview of the roles of the SUMO machinery throughout the intraerythrocytic life cycle of *Plasmodium* in the human host. **(A)** Before the first conjugation, SUMO is processed proteolytically by exposing its di-glycine motif at the C-terminal. SUMO in its mature state is activated by the heterodimeric enzyme E1 (AOS1-UBA2) in an ATP-dependent reaction, carried out by the AOS1 portion, which results in a thioester bond between the di-Glycine residue and catalytic cysteine in UBA2 (I). SUMO is then transferred to the catalytic residue of the enzyme E2 UBC9 (II). Finally, an isopeptide bond is formed between the Gly C-terminal residue of SUMO and a lysine residue on the substrate, generally supported by an E3 ligase (III) (Reverter and Lima, 2004). The SUMO deconjugation is catalyzed by specific isopeptidases/proteases that cleave the isopeptide bond between the SUMO C-terminal glycine and the lysine chain in the target proteins, restoring the mature SUMO for another cycle of conjugation (Ponder et al., 2011). **(B)** The roles of the SUMO machinery during asexual replication in erythrocytes. During asexual replication, merozoites infect erythrocytes and become intracellular ring forms, trophozoites, and schizonts that disrupt erythrocytes. The released merozoites can then infect new erythrocytes (Absalon et al., 2018). The so far explored molecular pathways which involves *Pf*SUMO, are transcriptional regulation (Tonkin et al., 2009; Absalon et al., 2018), oxidative stress response (Reiter et al., 2016) and ubiquitination/proteasome (Ponts et al., 2011) as presented in **Figure 1**. SIR2A is a *Pf*SUMO conjugate as validated by *in vitro* experiments (Tonkin et al., 2009).

Specifically, during *Plasmodium* SUMO conjugation, the heterodimeric *Pf*SUMO-E1 adenylates and exposes the mature C-terminal di-glycine motif of SUMO, *via* ATP-consuming reaction (Reverter and Lima, 2004; Reiter et al., 2013; Purushottam et al., 2019), **Figure 1A**. Then, mature SUMO is loaded into an internal cysteine residue of Uba2, forming a high-energy thioester bond (Reiter et al., 2016). This SUMO-E1 thioester heterodimeric enzyme is thereafter competent to recognize and enforce the SUMO transfer to the *Pf*SUMO-E2 enzyme (Reiter et al., 2013), forming a thioester linkage with the E2-catalytic (Hann et al., 2019). In the final step, the charged SUMO-E2 thioester complex can either directly load a SUMO unit to a lysine amino acid of the target substrate through the attachment of E2 in consensus SUMOylation motifs (Reiter et al., 2013; Streich and Lima, 2014) or mediate the SUMO transfer in an E3 ligases-dependent manner, such as *Pf*PIAS-E3 ligase (Reiter et al., 2013; Jain et al., 2017; Green et al., 2020) (**Figure 1A**).

The global SUMOylation levels are balanced and tightly regulated by reversible and highly dynamic SUMO-conjugation and SUMO-deconjugation events (Ponder and Bogyo, 2007). The SUMO deconjugation/removal is catalyzed by SUMO-specific isopeptidases/proteases that cleave off the isopeptide bond between the C-terminal glycine of SUMO and the lysine chain of target proteins, generating SUMO mature for another conjugation cycle (Rayavara et al., 2009; Ponder et al., 2011; Reiter et al., 2013). The *P. falciparum* genome encodes two deconjugases or deSUMOylating isopeptidase enzymes, namely, SUMO-specific protease (SENP) *Pf*SENP1 and *Pf*SENP2 (Wu et al., 2003; Ponder and Bogyo, 2007; Issar et al., 2008). While *Pf*SENP2 remains uncharacterized, *Pf*SENP1 was shown to be functionally active *in vitro* (Ponder et al., 2011). In this regard, it was demonstrated that *Pf*SENP1 can cleave the human SUMO-1 from the lysine motives of modified RanGAP1 and process SUMO precursors *in vitro* (Ponder et al., 2011).

## Roles of the SUMOylation Machinery Within the Life Cycle of *P. falciparum*

During the *Plasmodium* life cycle, sporozoites are deposited in the dermis of the host from the bite of infected female *Anopheles* and reach the bloodstream to migrate to the liver where they invade hepatocytes (Dundas et al., 2019; Singh et al., 2021). Sporozoites replicate inside hepatocytes and manipulate their environment to ensure the safe release of merozoites into the bloodstream through merosomes vesicles that project into the liver sinusoid capillaries (Sturm et al., 2006). Once released, merozoites invade red blood cells (RBCs) and initiate the asexual blood stage of infection, which is associated with clinical symptoms of malaria (Vijayan and Chitnis, 2019; Matz et al., 2020), **Figure 1B**. Inside of the erythrocytes, the parasite remodels the host cells and form the parasitophorous vacuole, which provides an environment for the development into intracellular forms from ring stages to trophozoites and then to schizonts, through a process referred to as asexual replication (Cowman et al., 2017; Matz et al., 2020), **Figure 1B**. *Plasmodium* schizogony starts with parasites shaped to a “ring” morphology, in which there is no DNA synthesis, then defined as interphase, G0 (Arnot et al., 2011). The progression to trophozoites is marked by the transition from the “ring” morphology into a spherical nucleus and is characterized by licensing of chromosome replication (G1 onset) and initiation of DNA synthesis (S phase) (Verma and Surolia, 2018). Following the replication, the schizont stage initiates with the single trophozoite nucleus dividing into two daughter nuclear bodies, followed by separation of the sister chromatids from the microtubule-organizing center (MTOC) - referred to as centriolar plaque - that assemble the mitotic spindle for chromosome segregation (M phase) (Gerald et al., 2011). The asexual life cycle culminates in the production of new merozoites, that rapidly egress from RBCs to invade new erythrocytes, which amplify exponentially the symptoms of malaria disease (Striepen et al., 2007; Absalon et al., 2018; de Jong et al., 2020; Molina-Franky et al., 2020), **Figure 1B**.

The complex life cycle of *Plasmodium* involving the progression through multiple morphologically and functionally distinct stages requires regulation by SUMOylation machinery. Biochemical, microscopy, and proteomic studies have been used to reveal the importance of SUMOylation machinery in regulating critical proteins and biological pathways that result in an efficient infection cycle in the mosquito and to human host cell invasion during the progression of the intra-erythrocytic life cycle of the parasite (Maruthi et al., 2017; de Jong et al., 2020), **Figure 1B**. In this regard, SUMOylation may enable routes to the parasite to convert in multiple stages in several hosts, cell types, and environments, implicating successful virulence and survival.

The importance of SUMOylation machinery for accomplishment the *P. falciparum* life cycle can be supported by the expression peak of *Pf*SENP1 and *Pf*SENP2 transcripts emerging approximately 25 hours post-invasion in the late trophozoite phase, while the lowest expression occurs in the early ring stage, immediately post-invasion (Bozdech et al., 2003; Le Roch et al., 2003). Similarly, fluorescence microscopy examination and biochemical assays on infected RBCs revealed that *Pf*SUMO or SUMO-conjugates were readily detectable with the highest levels reaching in trophozoite and schizont stages and being lowest in ring-stage parasites (Reiter et al., 2013) (**Figure 1B**). Further, a selective and potent inhibitor of *Pf*SENP1 activity, VEA-260, alters late schizonts morphology and disables parasite rupture from the RBC, supporting that blocking in SUMOylation machinery may be detrimental for the parasite releasing from the host cell (Ponder et al., 2011). Thus, SUMOylation may represent a key mechanism that malaria parasites use to perpetuate the life cycle in the host.

Notably, relevant proteins have emerged as potential *Pf*SUMO substrates and/or interactors during the red blood cell cycle of *P. falciparum* (Issar et al., 2008), **Figure 1B**, becoming evident the SUMOylation is a key event for the parasite’s life cycle. Among the biological pathways by which *Pf*SUMO substrates and/or interactors are involved, the transcriptional regulatios (Absalon et al., 2018), oxidative stress responss (Reiter et al., 2016), ubiquitylation, and proteasome pathways (Ponts et al., 2011) are currently the most known. *Plasmodium* parasites require a dynamic protein repertoire and ubiquitous control of gene expression to accomplish their complex tasks (Issar et al., 2008). This has been evidenced by reports showing that *Pf*SUMO was immunostained at distinctive subcellular forms of the ring, trophozoite, and schizont stages during the intra-erythrocytic developmental cycle, in which it also immunoprecipitated with *Pf*SIR2, a nuclear protein implicated in the control of gene expression (Issar et al., 2008; Tonkin et al., 2009), **Figure 1B**.

During the late stages of the intra-erythrocyte cycle, malaria parasites break down hemoglobin into amino acids and toxic-free heme, leading to the generation of elevated levels of reactive oxygen species including superoxide and hydrogen peroxide (Becker et al., 2004). In this respect, it is reasoned that *Pf*SUMO may play an important protective role against oxidative stress induced by hemoglobin degradation during dynamic *P. falciparum* trophozoite and schizont stages (Reiter et al., 2016). *Pf*SUMO conjugation increased when *P. falciparum* trophozoites were treated with high doses of artemisinin, an antimalarial drug that promotes parasite killing by overloading its oxidative stress response pathways (Reiter et al., 2016; Rocamora et al., 2018). Consistently, *Pf*SUMO was downregulated upon treatment of *P. falciparum* with the isocryptolepine derivative ICL-M, which exhibited antimalarial activity against trophozoites, due presumably to interference of their ability to generate an efficient stress response *via* SUMO (Rujimongkon et al., 2019).

Among post-translational regulations throughout the progression of the parasite’s life cycle, like SUMOylation, ubiquitination is certainly one of the most abundant (Green et al., 2020; Li et al., 2021). Ubiquitin is functional and conserved in *Plasmodium* when compared with other eukaryotes (Aminake et al., 2012). *P. falciparum* ubiquitylation itself plays an essential role in the development of transition from intracellular schizont to extracellular merozoite stages of the parasite (Green et al., 2020). Evidence indicates that SUMO/UB may function in important biological pathways in which ubiquitination is believed to label SUMOylated proteins to proteasomal degradation and serve as main regulators of cellular stress responses in *P. falciparum* (Ponts et al., 2011; Aminake et al., 2012). Notably, the synergism between ubiquitination and SUMOylation cascade is relevant to regulate the parasite’s life cycle (Shinde et al., 2020). Recent studies revealed that mutations in *Pf*SUMO-E2, *Pf*Uba2, *Pf*SUMO-E3 ligase, ubiquitin-activating enzyme 1, and E3-ubiquitin ligase are associated with high-level resistance to benzoxaborole AN13762. This compound is implicated in the loss of development of parasites from ring to trophozoite stages (Shinde et al., 2020). In this respect, ubiquitination/SUMOylation cascades following downstream proteasomal pathway activation may contribute to parasite virulence by regulating the protein turnover (Muñoz et al., 2015).

Taken together, these studies support that SUMOylation may play unique roles in the progression of the *Plasmodium* life cycle and highlight that SUMOylation machinery proteins as novel potential targets for antimalarial drugs. Although proteins modified by SUMO were described, the outcome of these modifications, as well as regulatory mechanisms in *P. falciparum*, remains largely uncharacterized. Specifically, the oxidative stress pathway has particular importance for the biology of parasites which ensures its relevance for future research towards new drugs for malaria treatment.

## SUMOylation as a Potential Target for Malaria Disease

From this perspective, it might be rationalized that the components of the SUMOylation machinery can become potential therapeutic targets in malaria. Here, we pinpoint SUMOylation machinery proteins as unexplored attractive new targets for the development of parasite-specific inhibitors and therapeutic intervention toward malaria disease.

As one of the main PTM pathways deregulated in human diseases, the interference of the SUMOylation modulation pathways, using experimental inhibitors, has been extensively attempted in mammalian cells (Brackett and Blagg, 2020). However, most of the current SUMOylation inhibitors are either peptidomimetics or originated from natural sources, with a rising number of small molecules being studied. Unfortunately, this investment has yet to be translated towards their *Plasmodium* homologs, where little drug discovery has been reported.

**Figure 2:**
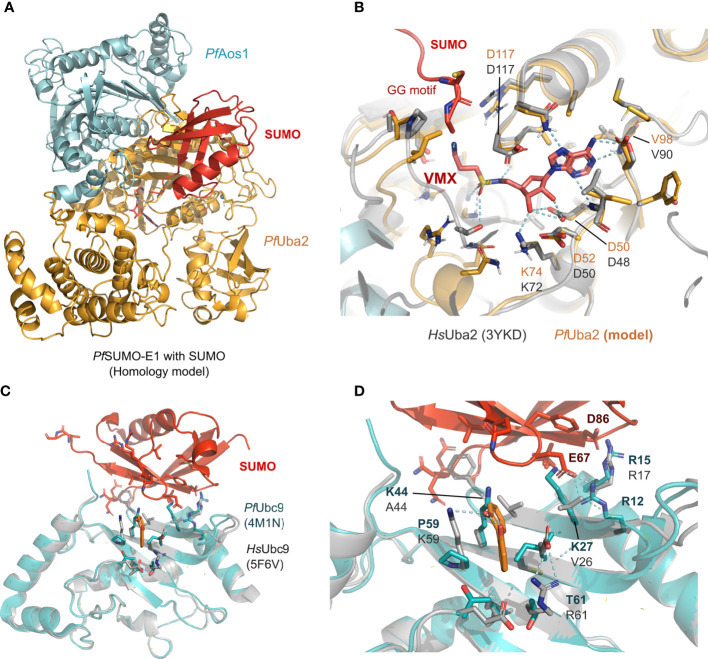
Structural models of the SUMO machinery highlight their folding conservation and high similarity in the active site. **(A)** overview of *Pf*SUMO-E1 complex modeled after the human homolog, where the interface between *Pf*Aos1 (cyan) and *Pf*Uba2 (orange) can be seen together with the interaction surface of SUMO (red). **(B)** *Pf*Uba2 catalytic site superimposed with the human Uba2 homolog (gray, PDB ID 3YKD), with conserved residues depicted as sticks, the analog of AMP-activated state (VMX) is shown to represent the substrate-binding site and its connection with the SUMO’s di-glycine motif. **(C)** SUMO-E2 machinery, represented by the *Pf*Ubc9 (in teal, PDB ID 4M1N) superimposed with the *Hs*Ubc9 (dark gray, 5F6V with its co-crystallized compound fragment in orange), highlighting one of the potential SUMO interaction surfaces described in the original crystal structure. **(D)** Allosteric fragment binding for the *Hs*Ubc9 (orange sticks), near the SUMO interface, but not conserved in the *Plasmodium* homolog. *Plasmodium* homology models were generated using I-Tasser and are available upon request. In all structural comparisons, superimposition between human crystal structures and *Plasmodium* homology models were generated by aligning their carbon alpha coordinates.

Currently, only the *Pf*SUMO-E2 crystal structure has been solved (Reiter et al., 2013). In terms of homology, the other two human and *Plasmodium* SUMOylation proteins share a distance relationship with E1/Aos1 registering 27%, E1/Uba2 - 33%, and the overall E3 - 28% sequence similarity. However, despite the low similarity, structural comparison between human SUMO-E1 and E2 and *P. falciparum* models suggests vastly conserved binding sites for SUMO and potential inhibitors (**Figures 2A–D**). Specifically, structural comparison between SUMO’s interaction surface from *P. falciparum* (herein represented by a homology model of the *Pf*Uba2, orange, in complex with the *Pf*Aos1, cyan, in **Figures 2A, B**) superimposed with the human crystal structure (represented in gray, **Figure 2B**) shows high conservation in all residues relevant for the ATP substrate and SUMO stabilization. The VMX small molecule, co-crystallized in the original human structure (PDB ID 3YKD), represents a mimetic of the intermediate state with the AMP portion and the di-glycine portion, which was employed to derive the active site position in the homology model from *Plasmodium* by superimposition. This suggests that targeting the classical active site of SUMO-E1 could lead to nonspecific inhibition and potential toxicity, despite the high druggability of this pocket. Prediction of potential druggable sites in the *Plasmodium* Uba2/Aos1 homology model (using SiteMap with standard options (Halgren, 2009), suggests a pocket extending from the VMX binding region towards the solvent-exposed surface, where the SUMO protein would couple with high SiteScores values (Supporting information, **Figure S1**). The SiteScore is employed to rank potential druggable sites in a protein, with values>0.8 standing for potentially interesting pockets (Halgren, 2009).

Alternatively, *Pf*Ubc9 is divergent from the human one, despite sharing the same folding (as suggested by the homology model generated in our group, **Figure 2C**). Previously described allosteric sites for the human Ubc9 (structurally defined in the PDB ID 5F6V) are also not conserved (Hewitt et al., 2016), as we observed by the superimposition against the *Plasmodium* model. This suggests that Ubc9 would be an interesting but challenging drug target since the potential binding sites are solvent-exposed. Prediction of potential druggable sites in the *Plasmodium* homology models showed three potential surfaces with moderate to low druggability scores (0.3-0.7), which highlights that this might be a challenging drug target (Supporting information, **Figure S1**).

In parallel to the main deSUMOylation machinery, targeting the SUMO deconjugating protease was shown to be effective in controlling *P. falciparum* replication. In particular, the *Pf*SENP1 inhibitor JCP-666 was identified from a library of irreversible cysteine proteases inhibitors against parasite lysates (Ponder et al., 2011). JCP-666 contains an aza-epoxide reactive group with a P1 aspartic acid side chain, which is typical for proteases. The aspartate moiety was later removed in recently generated analogs, such as VEA-260, to improve chemical stability without compromising target activity, remaining active even on a recombinant level. VEA-260 was also shown to be a PfSENP1 inhibitor and specifically among falcipain 1/2/3 proteases. However, further development would be required to achieve *Plasmodium* parasite selectivity since it presented some degree of activity against human SUMO-deconjugases [1,2 and 8, but not 6 or UCH-L3 (Ponder et al., 2011)].

## Author Contributions

EES conceived and wrote the majority of the manuscript. DS and TK performed the figures and wrote the original draft. CW and GP contributed to manuscript revision. All authors contributed to the article and approved the submitted version.

## Funding

The authors acknowledge the financial support provided by Fundação de Amparo à Pesquisa do Estado de São Paulo (FAPESP) *via* the grants 2015/26722-8, 2017/03966-4, 2018/10150-3, 2018/18257-1, 2018/15549-1, 2019/26771-0, 2020/12277-0, 2020/04923-0 and by Conselho Nacional de Desenvolvimento Científico e Tecnológico (CNPQ) through the grant 301524/2019-0.

## Conflict of Interest

The authors declare that the research was conducted in the absence of any commercial or financial relationships that could be construed as a potential conflict of interest.
