# Supplementary material for: Targeting SUMOylation in Plasmodium as a Potential Target for Malaria Therapy
Source: Front Cell Infect Microbiol. 2021 Jun 10;11:685866. doi: 10.3389/fcimb.2021.685866 (PMC8224225; doi:10.3389/fcimb.2021.685866)
Supplement: Supplementary file 1 [file DataSheet_1.docx]

Targeting SUMOylation in *Plasmodium* as a potential target for malaria therapy

Daffiny Sumam de Oliveira^1^, Thales Kronenberger^2^, Giuseppe Palmisano^1^, Carsten Wrenger^1^, Edmarcia Elisa de Souza^1*^

**SUPPORTING INFORMATION**


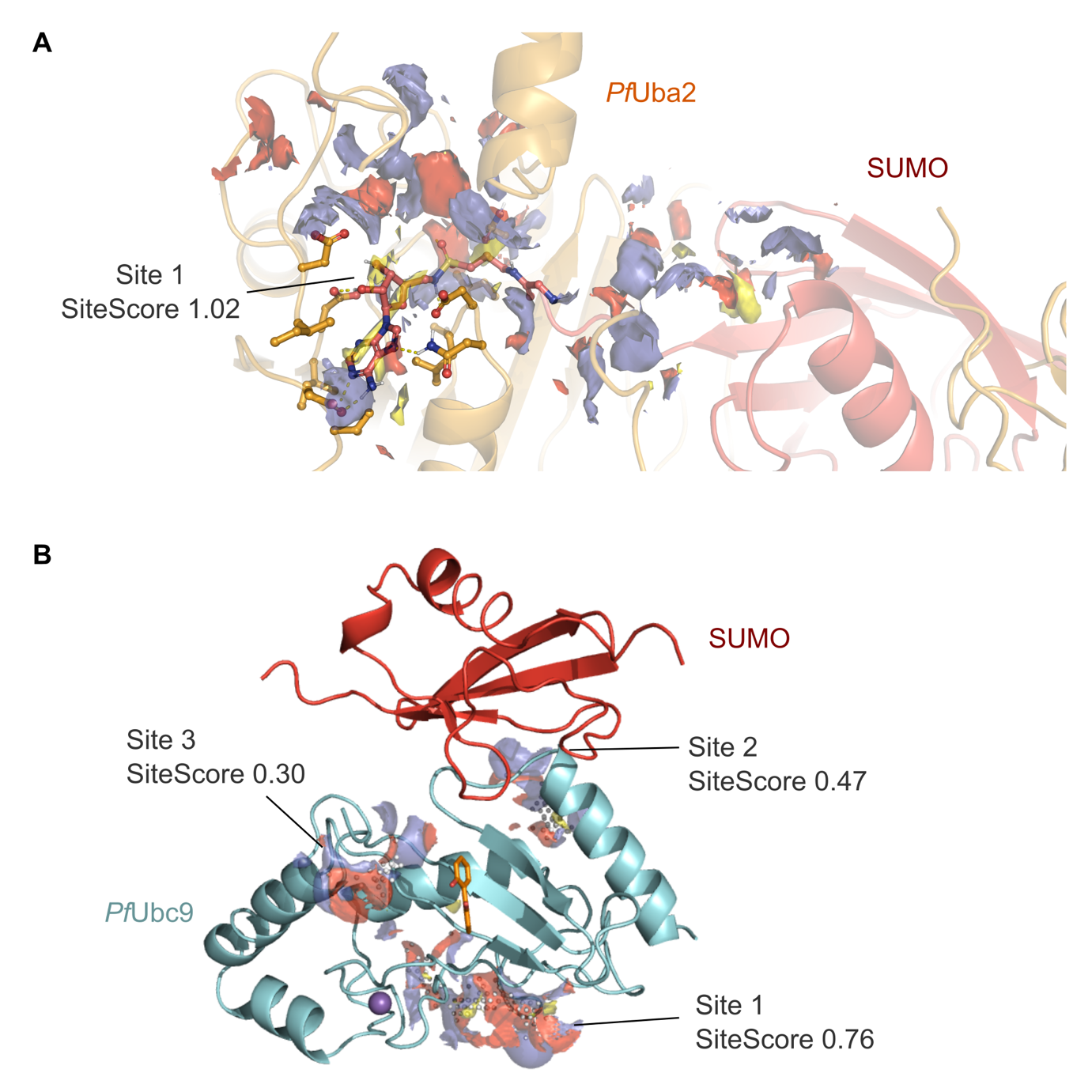


**Figure S1. Top ranked predicted druggable pockets using SiteMap tool. (A)** Figure highlighting the highest scored predicted druggable pocket in the *Pf*Uba2 catalytic site. This was superimposed with the analog of AMP-activated state (VMX), which is shown to represent the substrate-binding site and its connection with the SUMO’s di-glycine motif (originally positioned from the human homolog structure (PDB ID 3YKD). **(B)** SUMO-E2 machinery, represented by the *Pf*Ubc9 (in teal, PDB ID 4M1N) highlighting the allosteric fragment binding for the *Hs*Ubc9 (orange sticks), near the SUMO interface, but not conserved in the *Plasmodium* homolog. All three predicted druggable surfaces are distributed along the *Pf*Ubc9 structure with moderate to low SiteScores and almost no enrichment no hydrophobic surfaces, which agrees with the solvent exposures of these pockets. *Plasmodium* homology models were generated using I-Tasser using standard options. Yellow surfaces represent regions where hydrophobic interactions are well accepted, red and blue surfaces represents hydrogen donor and acceptors, respectively. SiteScore values higher than 0.8 suggests potentially druggable pockets.
